# Multi-level mental health-related stigma interventions in healthcare systems through the involvement of people with lived experiences: Mixed-methods pilot findings from international study of discrimination and stigma outcomes (INDIGO) study in Nepal

**DOI:** 10.1371/journal.pmen.0000638

**Published:** 2026-09-08

**Authors:** Bhawana Subedi, Kalpana Bhattarai, Brandon A. Kohrt, Claire Henderson, Elaine Brohan, Graham Thornicroft, Ioannis Bakolis, Maya Semrau, Nicole Votruba, Petra C. Gronholm, Uta Ouali, Dristy Gurung

**Affiliations:** 1 Transcultural Psychosocial Organization Nepal (TPO), Kathmandu, Nepal; 2 University of New Haven (UNH), West Haven, Connecticut, United States of America; 3 Center for Global Mental Health Equity, Department of Psychiatry and Behavioral Health, George Washington School of Medicine and Health Sciences, Washington, District of Columbia, United States of America; 4 Health Service and Population Research Department, Institute of Psychiatry, Psychology and Neuroscience, King’s College London, London, United Kingdom; 5 Centre for Global Mental Health, Institute of Psychiatry, Psychology and Neuroscience, King’s College London, London, United Kingdom; 6 Centre for Mental Health Policy and Evaluation, Health Service and Population Research Department, Institute of Psychiatry, Psychology and Neuroscience, King’s College London, London, United Kingdom; 7 Centre for Equitable Global Health Research, Department of Global Health and Infection, Brighton and Sussex Medical School, University of Brighton and University of Sussex, Brighton, United Kingdom; 8 Nuffield Department of Women’s & Reproductive Health, University of Oxford, Oxford, United Kingdom; 9 The George Institute for Global Health, Imperial College London, London, United Kingdom; 10 Centre for Global Mental Health, Department of Population Health, London School of Hygiene & Tropical Medicine, London, United Kingdom; 11 Razi University Hospital, La Manouba, Tunisia; 12 Faculty of Medicine of Tunis, University of Tunis El Manar, Tunis, Tunisia; National University of Singapore, SINGAPORE

## Abstract

Mental health-related stigma is a barrier to access to mental health services. This mixed-methods pre-post pilot feasibility study aimed to adapt and pilot-test a multi-level anti-stigma intervention across three levels of the health system to assess its feasibility and potential to reduce mental health-related stigma in Nepal. Contact-based interventions integrating people with lived experience in mental health and stigma training packages were adapted and implemented, targeting female community health volunteers (n = 19), primary healthcare workers (n = 9), and psychiatrists and psychiatric nurses (n = 9). Stigma-related knowledge, attitudes, and competency were measured pre- and post-training with follow-up at 3 and 6 months. Qualitative interviews were conducted with 43 participants across levels. In INDIGO Local, knowledge score increased from 20.43 (95% CI: 20.0-20.9) at pre-training to 25.11 (95% CI: 23.83-26.17) at six months. In INDIGO Primary, the intended behavior increased from 16.67 (95% CI: 14.73-19.27) at pre-training to 18.88 (95% CI: 17.69-20.31) at post-training, while SDS mean score decreased from 39.26 to 20.62. The PCP’s helpful behavior increased, and harmful behavior decreased. The knowledge and OSCE mean score in INDIGO READ-MH increased from 12.67 (95% CI: 10.36 -14.97) at baseline to 14.67 (95% CI: 13.28-16.05) at the post-training and from 2 (95% CI: 1.62-2.38) at pre-training to 9 (95% CI: 7.56-10.44) at post- training, respectively. The ASTAD subscale increased at three months, except for motivation and work satisfaction, which decreased at post-training. Qualitative data highlighted the positive attitudinal shift following the interventions, with strong emphasis on PWLE involvement. Overall, the providers perceived the training as feasible and appropriate within Nepal’s health system. However, whilst they reported more positive perceptions towards persons with mental health conditions following the interventions, they also identified barriers to effective mental health care, such as insufficient availability of medicines, limited human resources, or separate counseling rooms.

## Introduction

Majority of people with mental health problems are residing in low- and middle-income countries (LMICs) with limited access to mental health care [1,2]. Although Nepal lacks evidence-based data on the burden of mental health conditions, a 2019 study stated approximately 3.9 million, representing 13.5% of the total Nepalese population, were living with mental health disorders such as anxiety and depression [3]. Despite this burden, there is a significant treatment gap in Nepal due to the persistent underlying mental health-related stigma with 9 of 10 people with mental health conditions not having access to care [4,5]. The stigma and discrimination embedded in policies and health systems further affects individual living in rural areas and those with severe mental health problems, especially in LMICs like Nepal, stigma acts as a major barrier to reducing the burden of mental health conditions and improving access to care [6].

People with lived experience (PWLE) of mental health conditions have referred to mental health stigma as more agonizing than the illness itself [7]. Healthcare providers are significant pillars of the health system, fostering people’s health by delivering effective and quality mental health services. However, several studies have shown that stigma enacted by healthcare providers is a major bottleneck in mental health service utilization [8–10]. Healthcare providers in Nepal feel threatened by PWLE (fear of harm), think that they have a lower status if associated with mental health patients (fear of status loss), or feel incompetent in dealing with mental health patients (fear of losing professional credibility) [11]. This might result in negative attitudes and behaviors towards PWLE, making the healthcare setting a prominent location of stigma manifestations [12].

Nepal, a LMIC in the South Asian region, faces several challenges, such as stigma at the health system level, in scaling up a community-based mental health program [13]. In recent years, Nepal has taken a step forward to improve mental health services by adapting and implementing the Mental Health Gap Action Program (mhGAP) [14], developed by the World Health Organization (WHO), in community healthcare settings. Nevertheless, the stigma associated with mental health persists as a significant demand and supply side barrier to making mental health services accessible [15]. Healthcare providers often endorse negative stereotypes such as that PWLE are violent or incurable, feel unprepared to manage people with such mental health problems, leading to fear, avoidance, or discriminatory behaviors [11,12]. Even at the tertiary level, mental health specialists are trained in a biomedical model and are often not prepared to identify or deal with the interpersonal and self-stigma challenges faced by PWLE during their recovery [9].

Hence, the International Study on Discrimination and Stigma Outcomes (INDIGO) partnership program aimed to develop evidence-based strategies to reduce mental health-related stigma, which were implemented in seven sites in five LMICs, including Nepal [16]. Although a range of anti-stigma interventions, particularly education and contact-based approaches, have demonstrated effectiveness in reducing mental health-related stigma, very few have been cross-culturally adapted and implemented in low-income settings [17]. Fewer still have systematically targeted multiple levels of the health system structure. This study, therefore, aimed to adapt and pilot-test anti-stigma interventions across three levels of the health system- community, primary care, and specialist care in order to assess their feasibility and acceptability which highlights the interventions effectiveness to address the mental health related stigma in Nepal and similar low resource settings.

## Methods

### Ethics statement

Ethical approval for this study was obtained from the PNM Research Ethics Subcommittee of King’s College London (HR-19/20–17252) and the Nepal Health Research Council (registration no: 355/2021). Written informed consent was obtained from all participants before their involvement in the training and baseline data collection. Moreover, the photo release consent form was signed by the PWLE and each person appearing in their photo story.

### Intervention components and adaptations

The INDIGO partnership program consisted of three packaged interventions (INDIGO Local, INDIGO Primary, and INDIGO READ-MH) targeting different cadres of healthcare providers within the mental healthcare system. The Reducing Stigma among Healthcare Providers (RESHAPE) training curriculum was the foundation for fostering social contact with PWLE [11,18]. In RESHAPE, PWLE are trained using PhotoVoice to tell recovery stories using photographic narratives. PWLE were trained over 12 weeks to build their capacity to develop and narrate their stories through Photovoice. They were taught on public speaking, disclosure, managing distress, and photography. They create a photo narrative that includes pictures of their experiences of mental health conditions, treatment, and recovery. RESHAPE also includes a curriculum for training aspirational figures who are primary healthcare workers who share positive narratives about engaging with PWLE, as well as myth-busting about common misconceptions related to mental health.

These packages of interventions and their adaptations to fit implementation in Nepal are described below.

i) INDIGO Local [19] aimed to raise mental health awareness and increase referrals of people with mental illness at the community level. The intervention was adapted for implementation with female community health volunteers (FCHVs), who are local community members primarily involved in preventative and promotional health activities in Nepal. The intervention was adapted to fit within the Nepal government’s four-day training package for FCHVs, which was focused on improving referrals to primary care from the community using the Community Informant Detection Tool (CIDT) [20]. Stigma components such as contact intervention through RESHAPE Photovoice recovery narratives [21] by PWLE, identifying and reducing community stigma, and myth busting exercises were added to the training package for FCHVs. Additionally, a community stakeholder workshop consisting of Local health and political leaders, teachers, and non-profit organization members was conducted to identify potential media campaign methods. Based on their recommendations, a community media campaign using flyers and hoarding boards placed in strategic locations (marketplaces, outside school premises, outside health posts and nearby municipality administrative buildings,) was implemented to raise awareness about mental health in the community.
ii) INDIGO Primary [22] aimed to reduce stigma attitudes of primary care providers’ (PCPs) and increase their competency to provide quality mental health services in primary healthcare settings. This intervention was conducted using RESHAPE: PWLE PhotoVoice recovery narratives, along with stigma didactics and an aspirational figure (i.e., someone who already pre-intervention provided exceptional mental health service at primary healthcare facilities), were integrated within the Nepal government’s adapted WHO mhGAP training package for primary healthcare workers. This component was conducted as part of extension of ongoing cluster randomized controlled trial of RESHAPE in Western Nepal [23].
iii) INDIGO READ-MH [24] targeted at the specialist level to positively respond to the stigma and discrimination affecting PWLE. We adapted the training package for to fit the cultural context of mental health patients visiting specialists at the hospital. Specialists receiving the 2-day training were psychiatric residents and nurses working in psychiatric wards of government hospitals and medical colleges in an urban setting in Nepal. The training focused on specialists recognizing their own stigma and its effects, identifying and responding to patients’ reports of stigma and discrimination, and fostering advocacy at individual, family, and service levels. The training consisted of didactic and video sessions, along with experience-sharing by mental health specialists and recovery narratives by PWLE through Photovoice sessions.

**Fig 1:**
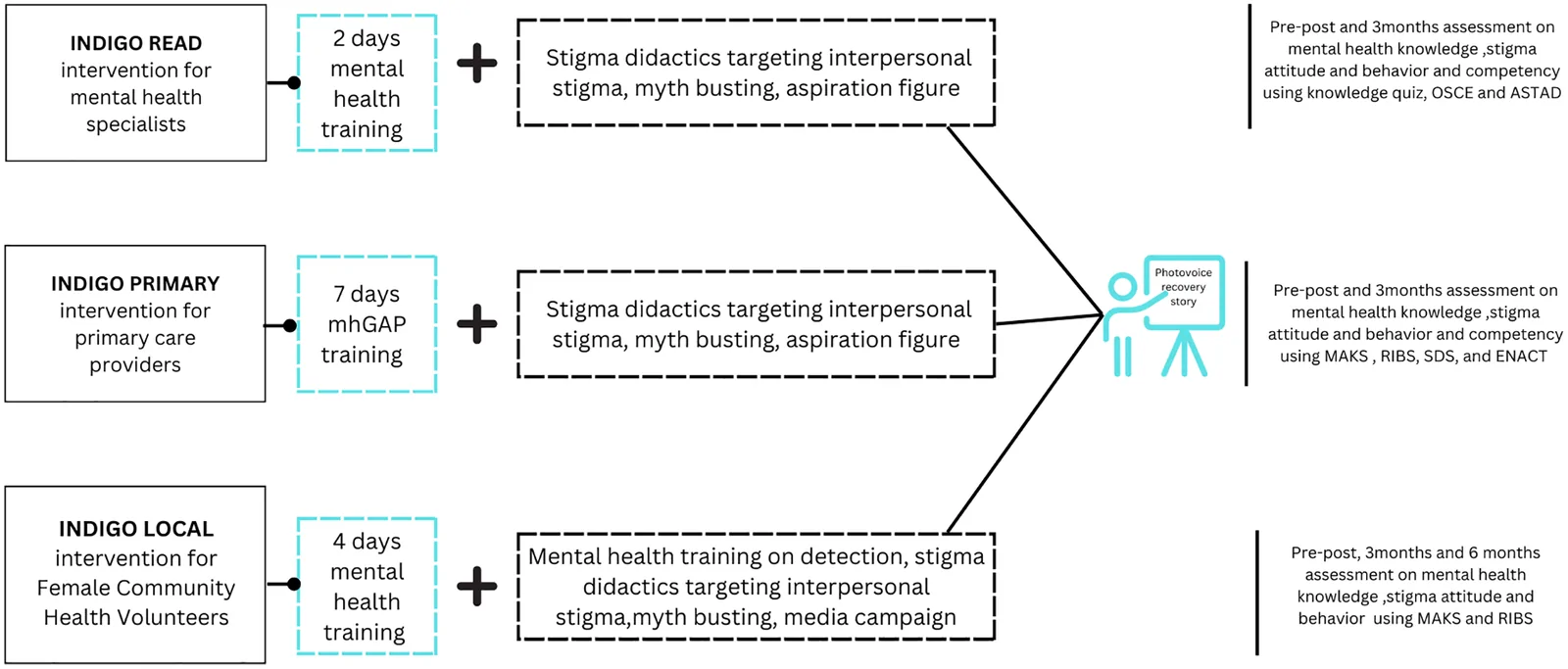
INDIGO programme intervention components as applied in Nepal. MAKS: Mental Health Knowledge Schedule, RIBS: Reported and Intended Behavior Scale, SDS: Social Distance Scale, ENACT: Enhancing Assessment of Common Therapeutic Factors, OSCE: Objective Structured Clinical Examination, ASTAD: Attitudes to Addressing Stigma and Discrimination Scale.

The intervention components of INDIGO Local, Primary, and READ-MH are explained in Fig 1 below.

### Study setting

In Nepal, the health system is structured into administrative tiers, with specialized healthcare services provided by the federal government through national hospitals. The provincial government governs the services provided by primary and secondary hospitals, while basic health services provided through primary healthcare facilities are overseen by municipalities. Until recently, mental health services were primarily delivered by a handful of private hospitals and medical colleges, while the only national specialist service was provided by a mental hospital located in the capital city, Kathmandu. However, due to the recent scale-up of the community mental health program, primary healthcare workers and community health volunteers in Nepal have started receiving a 3–5 days training package. It is within this setting that the INDIGO partnership program was implemented in Nepal. INDIGO Local and Primary were conducted in the Arjunchaupari Rural Municipality of Syangja District in the Gandaki Province, located in Western Nepal. Arjunchaupari is a rural municipality with four health posts serving a catchment population of 16,680. INDIGO READ-MH was conducted with specialists based in Pokhara Metropolitan City, Gandaki Province. Pokhara has four teaching hospitals providing mental health care, along with a tertiary regional hospital with a psychiatric department.

### Study design

This was a mixed-methods pre-post feasibility study that evaluated the anti-stigma interventions aimed to reduce mental health-related stigma at three levels of the healthcare system (community, primary, and specialist). INDIGO Local was implemented from 13^th^ September 2022–3^rd^ May 2023, INDIGO Primary from 3^rd^ March 2022–27^th^ November 2023, and INDIGO READ-MH from 19^th^ January 2023–25th April 2023. Quantitative data were collected at three time points for each of these: before the intervention, immediately after the intervention and at three month follow up. For INDIGO Local, data were also collected at six month follow up. Qualitative data were collected through interviews conducted at the final time-point of each intervention.

### Participant recruitment

Since this was a feasibility study, purposive sampling was used to determine the sample size for INDIGO Local, Primary and READ-MH.

**INDIGO Local:** FCHVs from all four health posts in Arjunchaupari Municipality were invited to participate in the training. For the stakeholder workshop, local municipal leaders, health coordinators, teachers, administrative officers, and representatives from NGOs were invited, as recommended by the municipality’s officials.

**INDIGO Primary:** All PCPs licensed as health assistants or auxiliary health workers and working in the four healthcare facilities (health posts or primary healthcare centers) from the Arjunchaupari Rural Municipality were invited to participate in the training.

**INDIGO READ-MH:** The medical colleges and regional hospital providing psychiatric services in Pokhara Metropolitan City were formally invited to participate in the training workshop. The hospital administrators were sent information about the study, and were asked to nominate resident psychiatrists, psychiatrists, and psychiatric nurses from their respective institutions to participate in the training.

### Data collection

#### Quantitative data collection.

For the quantitative data collection, various tools were used to assess participants’ knowledge of stigma, attitudes, and behaviors, as described in Table 1. The locally adapted tools were used and the research assistants were trained before the data collection in data handling and error checking to ensure consistency and data quality. Moreover, regular supervision from the research team lead on data entry, data management was done.

**Table 1:** Quantitative tools used among the three tiers of healthcare providers.

S.N.	Administered Tools	INDIGO Intervention	Timeline
1	Mental Health Knowledge Schedule (MAKS) measures stigma-related mental health knowledge. It is a 12-item scale scored on a five-point Likert scale, where a higher score indicates higher knowledge [26].	INDIGO Local and INDIGO Primary	Pre-training, post- training, 3 months; 6 months for INDIGO Local
2	Reported and Intended Behavior Scale (RIBS) measures reported and intended stigmatizing/discriminatory behaviors towards people with mental health conditions. It is an 8-item scale where a higher score indicates the willingness to interact with a person with lived experience of a mental health condition [27].	INDIGO Local and INDIGO Primary	Pre-training, post- training, 3 months; 6 months for INDIGO Local
3	Enhancing Assessment of Common Therapeutic Factors (ENACT) measures clinical competency. It has 18 items, and the competency of an item is based on a score of 2 or 3 on a 3-point scale [25].	INDIGO Primary	Pre-training, post-training, and 3 months
4	Social Distance Scale (SDS) measures willingness to engage with PWLE. The Nepali version has 12 self-report questions on a scale of 1–6 and three vignettes (depression, psychosis, and alcohol use disorder) [11].	INDIGO Primary	Pre-training, post-training, and 3 months
5	Knowledge Quiz assesses knowledge about source and impact of stigma and role to reduce stigma [24].	INDIGO READ-MH	Pre-training, post-training, and 3 months
6	Objective Structured Clinical Examination (OSCE) assesses the skills of specialists in identifying and addressing the impact of stigma and avoiding stigmatizing behaviors towards patients [24].	INDIGO READ-MH	Pre-training and post-training
7	Attitudes to Addressing Stigma and Discrimination Scale (ASTAD) assesses specialists’ attitudes towards addressing the stigma and discrimination faced by patients. The sum of items 1 and 2 denotes role adequacy, 7 and 8 denotes role legitimacy, 5 and 6 denotes motivation, 3 and 4 denotes task-specific self-esteem, 9 and 10 denote work satisfaction; the sum of role adequacy and legitimacy denotes role security, and the sum of motivation, work satisfaction and task- specific self-esteem denotes therapeutic commitment [24].	INDIGO READ-MH	Pre-training, post-training, and 3 months

**INDIGO Local:** The FCHVs completed a paper and pen-based evaluation form immediately before and after training, and at three- and six-month follow-ups before their group supervision.

**INDIGO Primary:** The PCPs’ data were collected at pre- and post-training time points, and after three months, before their group supervision. The stigma, knowledge, and attitude questionnaire was collected through the RedCap platform on a tablet. Trained counsellors evaluated clinical competency through the EQUIP-ENACT platform [25].

**INDIGO READ-MH:** The mental health specialist data were collected through both the tablets and ratings by experienced researchers pre- and post-intervention.

#### Qualitative data collection.

Thirty-two in-depth individual interviews (17 for INDIGO Local, 6 for INDIGO Primary, and 9 for INDIGO Read-MH), 6 PWLE and 5 Stakeholders, along with one focus group discussion (FGD) among the FCHVs were conducted by trained female researchers (BS, who was a BPH graduate, DG who was a Ph.D. fellow and KB was a BSc Biology graduate at the time of study implementation). DG has prior experience leading, analyzing, and publishing a qualitative study, and BS and KB had received qualitative training before the interviews. Semi-structured interview guides exploring the barriers and facilitators to the implementation of INDIGO Local, Primary, and Read-MH were developed and shared by the intervention leads and were translated and contextually adapted in consultation with Nepal site lead DG for the interviews. The in-depth interviews were conducted at convenient locations for participants, including the local community hall and a vacant room inside the hospital, with only one interviewer and participant present. Similarly, FGD was conducted at a health facility’s hall where one facilitator and a moderator from research team alongside participants were present. The in-depth interviews lasted 30–60 minutes on average while the FGD was approximately 50 minutes long. The interviews and FGDs were audio-recorded, and the researchers filled out debriefing forms after each interview to reflect on the key observations. Four participants were unable to participate in in-depth interviews due to their time constraints. The COREQ Checklist (S1 File) is included as a supplemental file describing the qualitative component.

### Data management and analysis

Quantitative data were collected and analyzed first to assess preliminary changes in knowledge, attitudes, and behaviors of the participants. Subsequently, qualitative data were collected through in-depth interviews and FDGs to further explore participants’ experiences, and to complement and provide deeper understanding of the quantitative results. Unique de-identifying code numbers were assigned to the participants to maintain their confidentiality.

### Quantitative data management and analysis

The quantitative data collected on paper (in INDIGO Local and READ-MH), the researchers manually entered the data in the RedCap software. For INDIGO Primary, the data were collected directly on RedCap through tablets. As this was a consortium-based project implemented in seven sites, data entered into RedCap from each site were centrally cleaned and then shared with the sites for site-based analysis. The cleaned Nepal-specific data were then analyzed by Local Nepali researchers using SPSS version 25. Descriptive summaries, change in mean scores pre- and post-intervention are reported.

### Qualitative data management and analysis

Audio recordings of the qualitative interviews and FGDs were transcribed and translated from Nepali to English by trained bilingual researchers. A thematic approach was used for qualitative data analysis [28] with the inclusion of both inductive and deductive approaches. A coding framework was generated using a priori themes from the interview guides as well as additional codes were generated from inductive coding of purposefully selected subset of interviews to ensure integration of any unanticipated themes. Three researchers independently coded the interviews and derived relevant codes until thematic saturation was reached, where no new themes emerged from the successive interviews and FDG [29]. This saturation approach is also described by the recent literature as the point where no any conceptual information arise from the data [30]. Moreover, iterative coding process and regular meetings with the coders was done to ensure addition of any new themes from the data. Some of the prior themes from the deductive framework were training experiences, including contents and methods, perception towards mental illness and people with mental illness, experiences of service users and aspirational figure training, and recommendations on training and interventions. The inductive themes derived from the interviews included service delivery barriers, media campaign process and barriers, and stigma encountered by professionals. The deductive and inductive themes were organized into four overarching themes: changes in knowledge, attitudes, and behaviors across intervention levels; feasibility and acceptability of implementing INDIGO interventions; multi-level collaboration across the intervention; and experiences of people with lived experience (PWLE) involvement. Dedoose version 9.2.12 was used for the coding and indexing of the data. Two coders (BS and KB) initially coded 4 interviews to refine the coding framework and establish inter-coder agreement. Any conflict in understanding between coders was discussed with the Nepal site lead (DG) after discussion.

## Results

A total of 39 participants were involved in INDIGO Local, Primary, and READ-MH at the pre-, 3-, and 6-month follow-ups (Table 2). The mean age across all three cadres was below 50 years, with the highest mean age observed in the INDIGO Local (44.7 years). The largest proportion of participants was from INDIGO Local among the three interventions, among which the INDIGO Local was entirely female (n = 21) and the INDIGO Primary and INDIGO READ-MH included both male and female participants. The participants were predominantly Brahmin across all three cadres, with Chettri representation in the INDIGO Local and READ-MH, and Madhesi participants in the INDIGO Primary. A small proportion of participants preferred not to disclose their ethnicity. The educational level of INDIGO Local was predominantly secondary-level, intermediate-level among INDIGO Primary, and master’s-level among INDIGO READ-MH participants. The professional roles were FCHVs in the INDIGO Local, auxiliary health workers and health assistants in INDIGO Primary, and psychiatrists, psychiatry residents, and nurses in INDIGO READ-MH.

**Table 2:** Socio-demographic characteristics of participants.

Characteristics	INDIGO Local (n = 21)	INDIGO Primary (n = 9)	INDIGO READ-MH (n = 9)
**Age**			
Mean (SD)	44.07(9.06)	29.78(6.74)	32(3.11)
**Gender, n (%)**			
Male	–	4 (44.4)	5 (55.6)
Female	21 (100)	5 (55.6)	4 (44.4)
**Caste, n (%)**			
Brahmin	13 (61.9)	4 (44.4)	4 (44.4)
Chettri	5 (23.8)	–	4 (44.4)
Dalit	–	1 (11.1)	–
Newar	–	–	–
Janajati	2 (9.5)	2 (22.2)	–
Madhesi	–	2 (22.2)	–
Prefer not to say	1 (4.8)	–	1 (11.2)
**Education, n (%)**			
Informal Education	–	–	–
Primary Education (Grade 1–5)	2 (9.5)	–	–
Lower Secondary Education (Grade 5–7)	2 (9.5)	–	–
Higher Secondary Education (Grade 8–10)	6 (28.6)	–	–
SLC	9 (42.9)	–	–
Intermediate (+2 passed)	–	9(100)	–
Bachelor’s level	–	–	–
Master’s level	–	–	9 (100)
Prefer not to answer	2 (9.5)	–	–
**Professional Role, n (%)**			
Female Community Health Volunteer	21 (100)	–	–
Auxiliary Health Worker	–	5 (55.6)	–
Health Assistant	–	4 (44.4)	–
MD General Practice	–	–	2 (22.2)
Psychiatry Resident	–	–	5 (55.6)
Psychiatric Nurse	–	–	2 (22.2)

### Changes on knowledge, attitudes, and behaviors across intervention levels

#### INDIGO Local.

**Fig 2:**
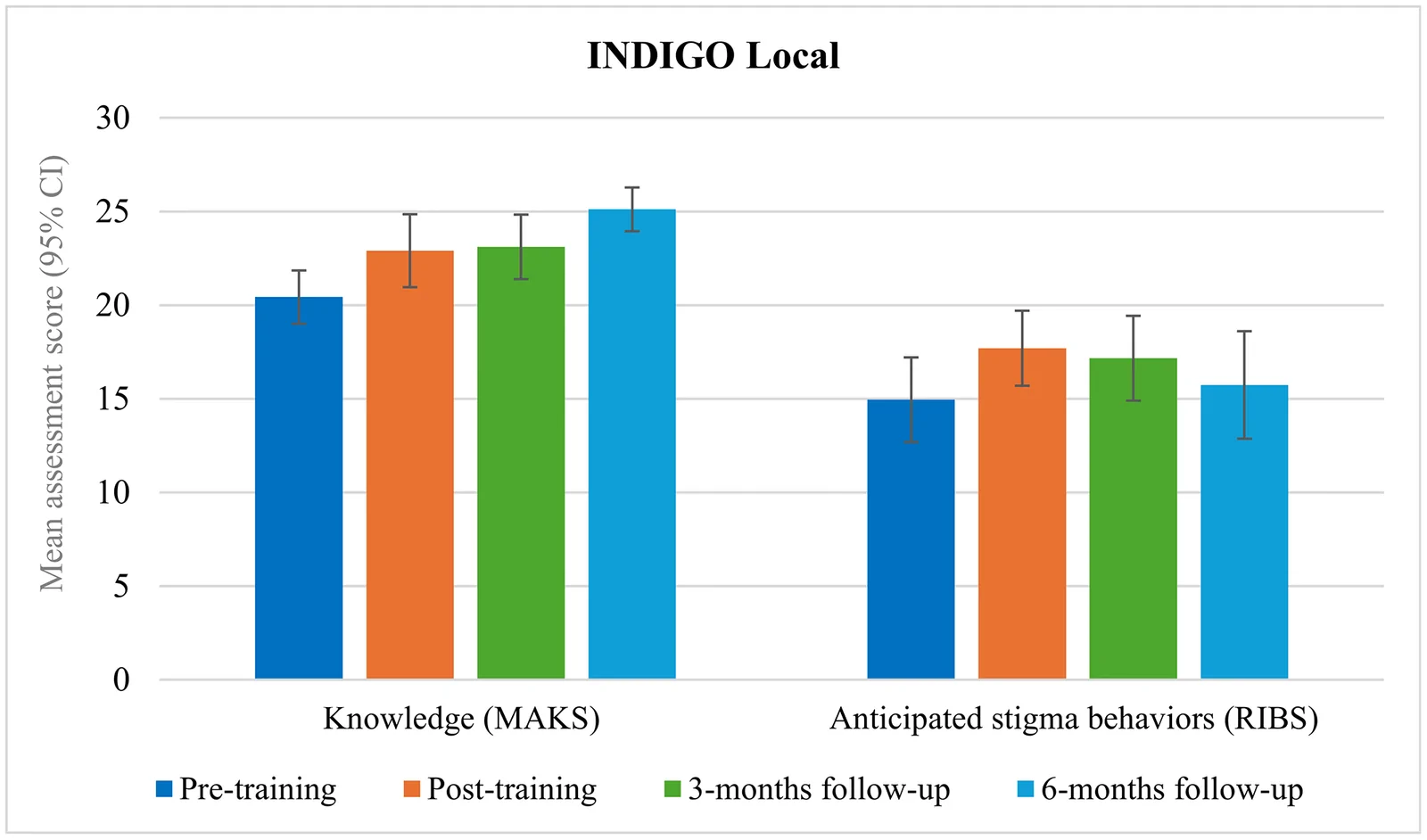
Stigma-related mental health knowledge and intended behavior among FCHVs pre, post, 3, and 6-month follow-up after the INDIGO Local intervention.

The stigma knowledge mean scores (measured using MAKS) were 20.43 at pre-training (95% CI: 20.0–20.9) and 22.90 at post-training (95% CI: 22.0–23.8) (Fig 2). At three months and six months, the mean scores were 23.11 (95% CI: 20.89-24.78) and 25.11 (95% CI: 23.83-26.17), respectively. The stigma behavior (as measured by RIBS) mean score increased from 14.95 (95% CI: 13.44-17.34) at pre-training to 17.70 (95% CI: 15.61-19.62) at post-training. The mean score declined to 15.74 (95% CI: 12.63-18.37) during 6 months follow-ups (Fig 2).

In the qualitative data, FCHVs expressed that, while the insights from the training helped them gain knowledge on mental health, frequent follow-up or booster interventions may be necessary to sustain the positive attitudinal gains. FCHV quoted;

*“So, it’s been six months of the training, but if that training happens frequently, we will also be reminded time and again of the subject matter. It makes us a little inactive waiting for six months.” (FCHV, Female)*

Moreover, the participants expressed that the lack of referral points after identification of people with mental health conditions in the community, stigma within the family or community, made it difficult for them to continue engaging and identifying cases in the community.

#### INDIGO Primary.

**Fig 3:**
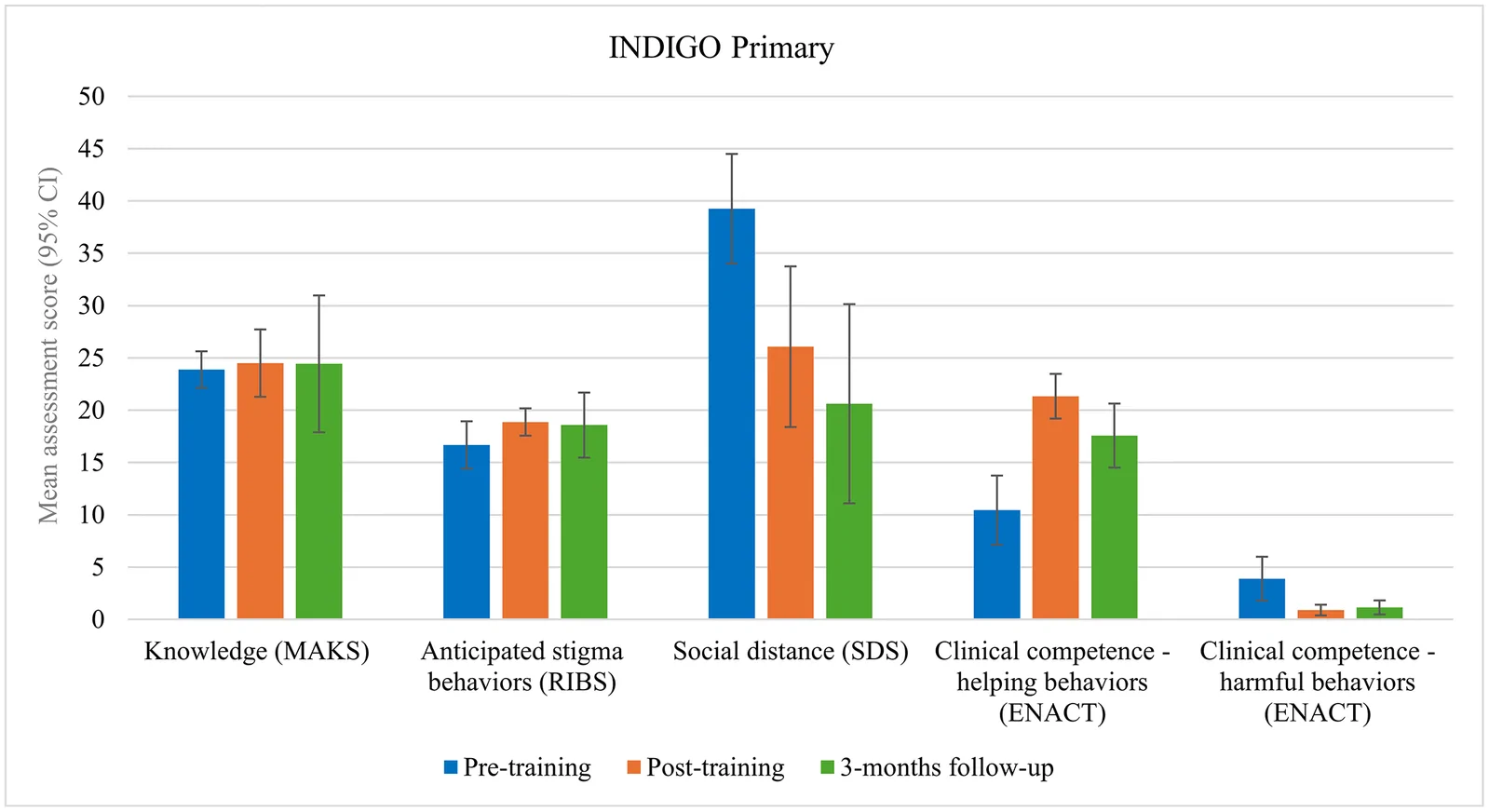
Stigma-related mental health knowledge, intended behavior, social distance toward PWLE, and clinical competency of PCPs pre-, post-, and 3 months follow-up after the INDIGO Primary intervention.

The stigma knowledge mean score of PCPs was 23.89 at baseline (95% CI: 22.15-25.63), 24.50 (95% CI: 21.28-27.72) during post-training, and decreased slightly to 24.43(95% CI: 17.89-30.97) at three months (Fig 3). The RIBS mean score increased from 16.67 (95% CI: 14.73-19.27) at pre-training to 18.88 (95% CI: 17.69-20.31) at post-training, with a slight reduction to 18. 57 (95% CI: 15.46-21.68) at three months. The mean SDS score decreased from 39.26 (95% 34.03-44.5) at pre-training to 20.62 (95% CI: 11.1-30.14) at the three-month follow-up (Fig 3). The mean score of helping behavior (Fig 3) among PCPs increased from 10.44 (95% CI: 7.13-13.75) at pre-intervention to 21.33 (95% CI: 19.19-23.47) in the post-intervention period, with a decline at three months to 17.57 (95% CI: 14.51-20.63). The harmful behavior of PCPs (Fig 3) decreased from a mean score of 3.89 (95% CI: 1.79-5.99) to 0.89 (95% CI: 0.38-1.40) at the post, with a slight increase of 1.14 (95% CI: 0.47-1.81) at 3 months.

In qualitative findings, the PCPs shared that in the past, they were hesitant to interact with patients with mental health conditions and referred them without proper examination, which had now changed to increase their confidence to deal with mental health patients in their routine practice.

“*There have been changes. We used to stigmatize them before; we used to tell them to stay away before. But now we stay together and chat with them. We listen to their difficulties or pain and give them counseling accordingly.” (Primary Care Auxiliary Health Worker, Female)*

The PCPs also acknowledged a shift from feeling incompetent in treating patients with mental health conditions to confidently providing counseling and treatment through health facilities after interacting with PWLEs and listening to their recovery narratives in the training.

*“Bringing real patients in front of us and letting them share their experience from the period before their illness, and during the treatment, and also what changes have come after the treatment, we were able to see these things by ourselves. After listening to the patient, my confidence increased even more.” (Primary Care Auxiliary Health Worker, Male)*

#### INDIGO READ-MH.

**Fig 4:**
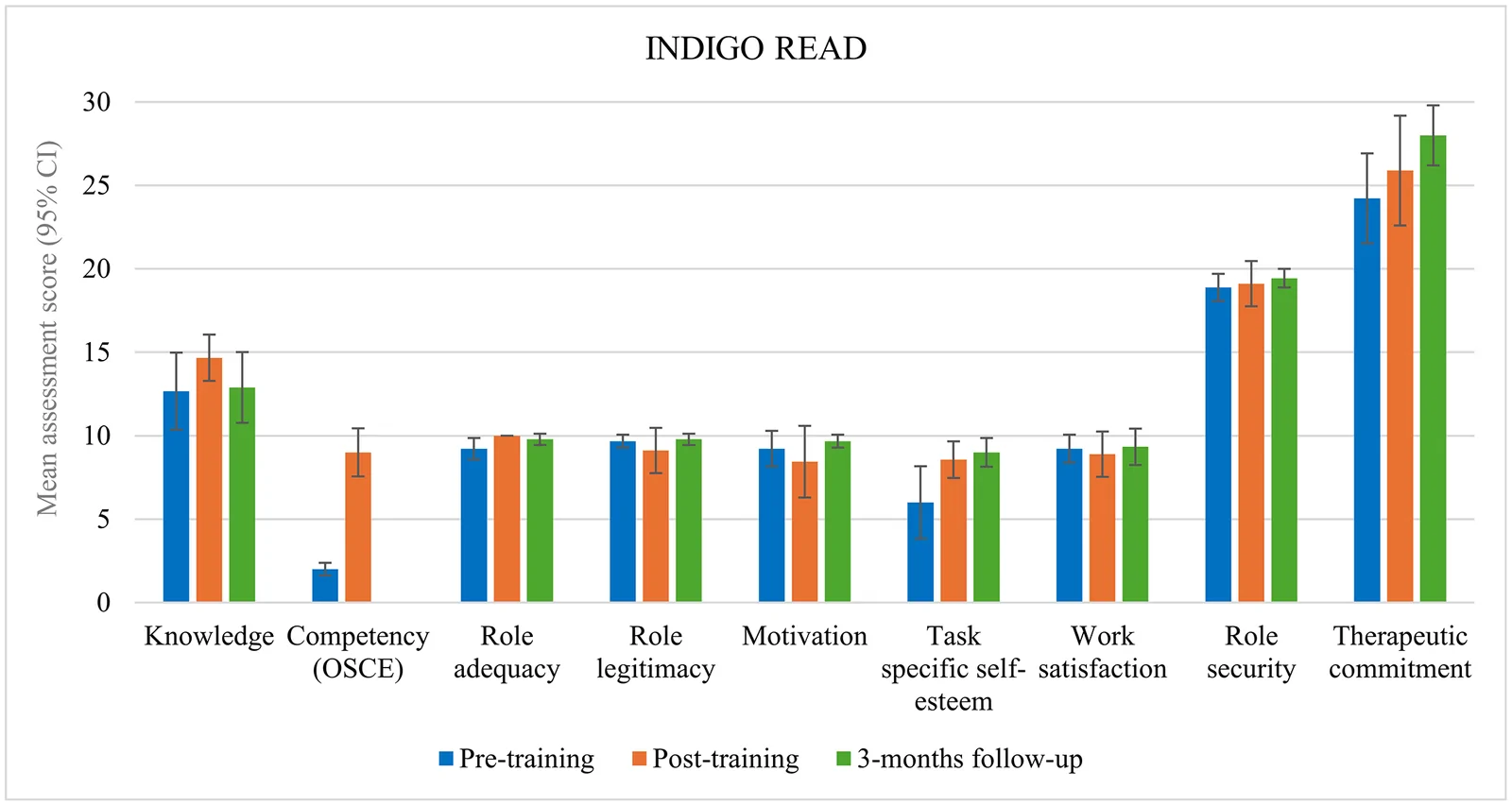
Knowledge, skills, and attitudes to addressing stigma and discrimination faced by patients with stigma among specialists, scores pre-, post-, and 3-month follow-up after the INDIGO READ-MH Intervention.

The results in Fig 4 indicate that the specialists knowledge mean score increased from 12.67 (95% CI: 10.36 -14.97) at baseline to 14.67 (95% CI: 13.28-16.05) at the post training (Fig 4). The knowledge score decreased over the three months to a mean of 12.89 (95% CI 10.77-15.01). The specialist’s skills (assessed through OSCE) (Fig 4) in identifying and addressing the stigma faced by patients, as measured by the mean score, have increased from 2 (95% CI: 1.62-2.38) at pre training to 9 (95% CI: 7.56-10.44) at the post-training (see Fig 4). The ASTAD role adequacy, task-specific self-esteem, role security, and therapeutic commitment showed increases in mean scores during the pre- and post-training periods and remained higher at 3 months (Fig 4). In contrast, role legitimacy decreased from 9.67 (95% CI: 9.28-10.05) at pre-training to 9.11 (7.76-10.47) at post-training and increased to 9.78 (95% CI: 9.44-10.12) at three months. Similarly, motivation and work satisfaction decreased during the post-test but increased at the 3-months follow-up (Fig 4).

During the qualitative interviews, specialists emphasized that they learned about various types of mental health stigma experienced by the patients through the Photovoice narratives of PWLE. They mentioned they started allocating more time to the patient and now probed more about any form of mental health stigma experienced by the patient after receiving the training.

*“Comparing the scenario before the training and after the training, before how the patients are considered the social context and family dynamics, how they are perceived in the society, and family dynamics as well were not considered by us. We would only look at what the illness was or what symptoms the person had. But now I have kept these aspects in priority as well.” (Psychiatry Resident, Male)*

### Feasibility and acceptability of implementing INDIGO interventions

Most participants of INDIGO Local, Primary, and READ-MH found the involvement of PWLE acceptable and appropriate in Nepal’s context for reducing stigma and improving mental healthcare at both community and hospital levels. Participants highlighted the added value of PWLE recovery narratives in evoking strong empathic feelings and increasing awareness of the effects of mental health stigma in service provision. One FCHV shared;

*“Her stories made me change my perception towards mental health. I found the involvement of the patient to be very effective. Before, I didn’t know anything about mental health. Now, I feel mental health problems can be cured, and people’s perceptions can also be changed, just like mine.”(FCHV, Female)*

Similarly, specialists stated they were completely unaware of the stigma and discrimination faced by PWLE prior to the training. That realization had led them to reflect upon the deeply rooted stigma within themselves and made changes in their workplace dynamics, such as nurses taking the detailed history of the patients, or ending the pattern of allocating the corner bed in the emergency ward to mental health patients. This shifted the focus on valuing the patients’ emotions and involving family members/caretakers in the treatment process after the training. A specialist shared:

*“Before, while working in the out-patient department (OPD), we used to rarely ask patients questions related to stigma and mental health. Now, we ask them about their family and how they are perceived in their family. I think this is somehow an improvement and we also feel we have changed ourselves in the way we ask questions to patients.” (Psychiatry Resident, Male)*

Nevertheless, participants mentioned a few challenges in attending the training. FCHVs trained within INDIGO Local and PWLE involved in all three interventions collectively mentioned that it was at times difficult to manage to attend the sessions whilst having to balance it with carrying carry out household chores. Additionally, the geographical terrains during the monsoon season made it challenging to attend the training.

In regard to the media campaign as part of the INDIGO Local intervention, a stakeholder (local leader) pointed out the limitations of the impact of flyers and hoarding boards for non-literate people and school children who cannot properly grasp the information. Additionally, given the lower socio-economic condition of people in the rural area, where people tend to seek traditional healing methods over spending money on mental health treatment, may take a long time to see the benefits of the media campaign.

*“It may not be helpful because this is a village area. Here, people are poor and financially weak; they think that getting treated will cost them a lot of money, so they believe in traditional methods like offering gods different things is one thing. The people here are not literate so it might take a lot of time to reduce or eliminate stigma and it is a gradual process.” (Stakeholder, Teacher, Female)*

In INDIGO Primary, although the intervention was deemed feasible and acceptable by the PCPs and PWLE, the frequent turnover of PCPs was a key barrier that caused disruption in mental health service delivery within primary healthcare facilities. Another key barrier for the implementation of INDIGO Primary was the COVID-19 pandemic, during which the training participation and service delivery were affected due to the halt of all other non-emergency services. PCPs’ workload was another barrier mentioned by participants. Due to high workload and shortage of staff in the healthcare facilities, the PCPs noted difficulties in engaging with mental health patients and providing mental health services, as this was more time-consuming than providing other physical health services.

*“Since, there are no employees, it was not possible to manage the time while everyone was doing the same thing, the way we should convince the patients, we could not counsel them according to the way we should, we could not give them accordingly, that’s a thing.” (Primary Care Auxiliary Health Worker, Male)*

For INDIGO READ-MH, specialists time unavailability for the training due to hospital work as well as the limited number of mental health specialists in the study site were barriers to intervention delivery. Hence, a mix of professionals such as psychiatric nurses and practicing psychiatrists were included along with resident psychiatrists. This limited the active involvement of all participants in the training due to the hierarchical nature of the positions that is seen in Nepal’s medical field. The resident psychiatrists were observed to be reluctant to share personal experiences of treating mental health patients and structural stigma within hospitals in the presence of the practicing psychiatrists. Similarly, nurses were also seen to be uncomfortable interacting with residents and practicing psychiatrists during the training. Moreover, a few specialists highlighted that the short training duration made them feel pressured to absorb a large amount of information within a limited time. A specialist shared:

*“As I already mentioned to you, the thing that needs to be improved is, in a short span, everything was discussed. If one or two days could have been added, the program would be more effective and easier for everyone.” (Psychiatrist, Female)*

The healthcare providers from INDIGO Local, Primary, and READ-MH also stressed community and structural stigma as barriers to delivering mental health services post-training. FCHVs shared difficulties in referring patients to health facilities because of the underlying mental health stigma of the patients’ family members, distrust in medication provided by the primary health center, and patients’ anticipated stigma, like fear of community people looking differently at them. The PCPs shared that not opening up to them about their problems due to a lack of a separate counselling room and distrust towards primary healthcare facilities due to the unavailability of medicine were some of the structural barriers and stigma limiting their work. This is stated by a PCP who participated in the INDIGO Primary training:

“*There was a challenge. Firstly, even if we took training and go to the health post, medicine did not arrive. First, we had to convince to the patient. After convincing, we become unable to provide 24 hours facilities. We also had to provide medication to patients and wait for how medication works and its effects. Patients would come but most challenging was unable to provide medication.” (Primary Care Auxiliary Health Worker, Male)*

Similarly, the specialists mentioned that although they were motivated to implement the learnings from the INDIGO READ-MH training, it was difficult for them to engage with patients and ask about stigma challenges due to the high volume of patients at tertiary hospitals.

*“I am trying to address that but it is not possible because of the volume of patients. We hardly are able to give 5-10 mins per patient. It’s not about here only it is not possible anywhere in Nepal, if you look at the doctors and patient ratio.” (Psychiatry Resident, Male)*

Despite the barriers, multiple facilitating factors supported the implementation of the interventions. Involving Local stakeholders in a stakeholder workshop prior to implementation led to buy-in and provided important contextual insights, resulting in the smooth implementation of the INDIGO Local and Primary interventions. The integration of stigma components into the government’s ongoing community mental health training packages was cited by participants as a sustainable and contextually relevant mechanism for delivering anti-stigma programs.

### Multi-level collaboration across the Intervention

Additionally, because both the INDIGO Local and Primary interventions were delivered in the same rural municipality, participants mentioned that it helped overcome some supply and demand barriers. The FCHVs were able to refer patients they identified locally to health facilities with PCPs trained in INDIGO Primary. Similarly, some of the barriers related to community stigma and structural stigma faced by PCPs while providing mental health services were mitigated by the stakeholder workshops and media campaigns run as part of the INDIGO Local intervention.

### Experiences of PWLE involvement

Although all levels of health cadres involved in the interventions acknowledged the positive effects of involving PLWE in reducing stigma, they also highlighted the possible risks of their involvement, such as increased stigma and family conflicts. The PWLE, however, acknowledged that the Photovoice training contents, such as safe disclosure, dealing with difficult questions, and involvement of caregivers throughout the process, and provision of travel allowances, facilitated their participation in the interventions.

*“My husband used to get irritated by my headache problem previously. After coming here with me, listening to all these things, he now understands the reason behind that. He now understands that this kind of problem has led to those symptoms. Those pains were because of my symptoms.” (PWLE, Female)*

## Discussion

This study explored the changes in stigma knowledge, attitudes and behavior of health cadres at three levels of the health system (community, primary, and specialists) through targeted anti-stigma interventions with strong contact-based components involving PWLE. The findings of this study indicate that the interventions were feasible and acceptable, with potential for positively changing the knowledge, attitudes, and behavior of the healthcare providers in Nepal through meaningful involvement of PWLE.

Among the three interventions (INDIGO Local, Primary, and READ-MH), the common approach to reducing stigma was a contact-based approach involving PWLE as facilitators in the health cadres’ training and stigma didactics (such as myth-busting exercises and understanding different types of stigma and their impact on patients). The involvement of PWLE was particularly valued across all groups and was seen as culturally appropriate and impactful in invoking empathy and changing perceptions about deeply embedded stigma rather than through didactic teaching. Photovoice narratives encouraged health cadres to reflect their own role in perpetuating stigma within the health systems, leading to shift such as reduced harmful behavior and improved helping behavior (engaging families, showing more respect, and providing counseling). Contact-based interventions to reduce stigma have been highlighted as the most effective method to tackle mental health stigma [7,31]. However, studies have warned about the negative effects of such contact on PWLE and have advocated preparing PWLE for the contact activities [32]. This was a crucial first step in the INDIGO interventions in Nepal, where the PWLEs underwent 12 sessions of Photovoice training which not only empower them to be educators to narrate their recovery stories via personal photos, but also addressed their key concerns, such as safe disclosure, handling difficult questions, and increase their confidence in public speaking. The training was based on the previous work of [33] on the use of Photovoice to reduce stigma within the healthcare system.

Factors such as stakeholder workshops for implementation planning, integration of stigma components into existing government training packages (for INDIGO Local and Primary), and implementation of the packages (INDIGO Local and Primary) in the same municipality made the interventions more feasible and acceptable. These strategies might promote these interventions as a permanent initiative rather than viewing it as a separate program. However, practical challenges, such as travel during the monsoon, household responsibilities of PWLE and FCHVs, COVID-19 disruptions, and structural barriers and stigma were identified as key barriers to implementation [4,34].

The unavailability of specialists in the rural municipality where INDIGO Local and Primary were implemented necessitated the implementation of INDIGO READ-MH in a different urban location. Hence, while patients identified by FCHVs trained within INDIGO Local in communities could be referred to the primary healthcare facilities where PCPs had received training under INDIGO Primary, leading to improved continuity of care, referrals of serious cases requiring specialist services could not be done effectively, leading to frustrations among PCPs and reduced sustained effects of the training [35,36]. Other structural barriers, including the lack of medicines at health facilities, inadequate counseling spaces, high PCPs turnover, and high workloads, were also cited as reasons why health cadres struggled to implement learnings from the INDIGO training effectively. The PCPs turnover also impacted the attitude and motivation of FCHVs within INDIGO Local, who were not able to refer identified cases to primary healthcare facilities. These persistent systemic barriers in long term may undermine the providers motivation to deliver mental health services, despite their positive shift in stigmatizing attitude and behavior.

This highlights the need for a systems approach to tackling stigma in mental healthcare rather than a siloed approach of focusing on individuals at the interpersonal level [6,37]. A framework such as FOCUS-MHS [38] which conceptualizes mental health stigma through a systems approach that involves multiple domains and actors, could help address the structural barriers and make such interventions more sustainable.

### Strengths and limitations of the study

This was a proof-of-concept study that demonstrated the feasibility of a multi-level stigma reduction intervention in a low-resource setting. The findings add to existing evidence that participatory and contact-based lived-experience-driven approaches can influence provider knowledge, attitudes, and behaviors [39,40]. Nevertheless, proper preparatory work and training of PWLE is a must prior to their involvement in such interventions to reduce any risks or harm. The study highlights the importance of adopting a cross-level (Local, primary, and specialist) and systems approach to tackling stigma to reduce barriers to implementation in future trials.

The study had its limitations. Since this was a small-scale proof-of-concept study, sample selection and sample size and stigma perspective of providers may have led to biased results, limiting the generalizability of the findings. The study, therefore, can be interpreted as trends rather than definitive evidence of effectiveness. Even though the study relies largely on self-report measures, the behavioral observational measures of clinical competence such as ENACT and OSCE) was a methodological strength of this study. The minimal changes in the role adequacy, legitimacy, motivation, satisfaction, and security at the post-intervention of INDIGO READ-MH suggest the practical application of training needed time to implement, as the data were collected right after the training, limiting the participants to integrate their learning into practice. Moreover, the tools used in quantitative data collection were locally adapted in consultation with the research team and experts from mental health background to fit the contextual relevance, although concerns remain regarding its validity and reliability. The short follow-up period (of 3–6 months), and disruptions to effective implementation (such as COVID-19 and workforce turnover) further limit the conclusions.

## Conclusions

This study demonstrated the feasibility and acceptability of implementing the INDIGO stigma-reduction interventions across the community, primary, and specialist levels of Nepal’s health system. However, the effects were not consistently sustained over time, and the results must be interpreted cautiously, considering the limited sample size. The implementation of the interventions was challenged by practical barriers (such as monsoon, transportation issues, and conflicting personal priorities) and structural and systemic barriers (such as workforce shortages, provider turnover, and limited resources), which constrained the translation of attitudinal shifts into sustained service improvements post-training. Nevertheless, embedding the interventions into existing training platforms and leveraging stakeholder engagement offered viable pathways towards sustainability and scale-up. Overall, the findings underscore the potential of participatory, multi-level anti-stigma approaches to strengthen mental health service delivery in low-resource settings. Future research should build on these insights through larger, adequately powered trials with longer-term follow-up, alongside strategies to address structural barriers, to better establish the effectiveness and scalability of the interventions.

## Supporting information

S1 File COREQ checklist. (DOCX)

## References

[1] Rathod S, Pinninti N, Irfan M, Gorczynski P, Rathod P, Gega L. Mental Health Service Provision in Low- and Middle-Income Countries. 10.1177/1178632917694350 PMC5398308 28469456

[2] Semrau M, Evans-Lacko S, Alem A, Ayuso-Mateos JL, Chisholm D, Gureje O, et al. Strengthening mental health systems in low- and middle-income countries: the Emerald programme. BMC Med. 2015;13:79. doi: 10.1186/s12916-015-0309-4 25879831 PMC4393637

[3] Dhungana RR, Pandey AR, Joshi S, Luitel NP, Marahatta K, Aryal KK, et al. The burden of mental disorders in Nepal between 1990 and 2019: Findings from the Global Burden of Disease Study 2019. Glob Ment Health (Camb). 2023;10:e61. doi: 10.1017/gmh.2023.55 37854421 PMC10579670

[4] Luitel NP, Jordans MJD, Kohrt BA, Rathod SD, Komproe IH. Treatment gap and barriers for mental health care: A cross-sectional community survey in Nepal. PLoS One. 2017;12(8):e0183223. doi: 10.1371/journal.pone.0183223 28817734 PMC5560728

[5] Luitel NP, Garman EC, Jordans MJD, Lund C. Change in treatment coverage and barriers to mental health care among adults with depression and alcohol use disorder: a repeat cross sectional community survey in Nepal. BMC Public Health. 2019;19(1):1350. doi: 10.1186/s12889-019-7663-7 31640647 PMC6806507

[6] Gurung D, Neupane M, Bhattarai K, Acharya B, Gautam NC, Gautam K, et al. Mental health-related structural stigma and discrimination in health and social policies in Nepal: A scoping review and synthesis. Epidemiol Psychiatr Sci. 2023;32:e70. doi: 10.1017/S2045796023000823 38086740 PMC10803190

[7] Thornicroft G, Sunkel C, Alikhon Aliev A, Baker S, Brohan E, El Chammay R, et al. The Lancet Commission on ending stigma and discrimination in mental health. Lancet. 2022;400(10361):1438–80. doi: 10.1016/S0140-6736(22)01470-2 36223799

[8] Carrara BS, Ventura CAA, Bobbili SJ, Jacobina OMP, Khenti A, Mendes IAC. Stigma in health professionals towards people with mental illness: An integrative review. Arch Psychiatr Nurs. 2019;33(4):311–8. doi: 10.1016/j.apnu.2019.01.006 31280773

[9] Henderson C, Noblett J, Parke H, Clement S, Caffrey A, Gale-Grant O, et al. Mental health-related stigma in health care and mental health-care settings. Lancet Psychiatry. 2014;1(6):467–82. doi: 10.1016/S2215-0366(14)00023-6 26361202

[10] Li J, Li J, Thornicroft G, Huang Y. Levels of stigma among community mental health staff in Guangzhou, China. BMC Psychiatry. 2014;14:231. doi: 10.1186/s12888-014-0231-x 25115221 PMC4149249

[11] Kohrt BA, Turner EL, Rai S, Bhardwaj A, Sikkema KJ, Adelekun A, et al. Reducing mental illness stigma in healthcare settings: Proof of concept for a social contact intervention to address what matters most for primary care providers. Soc Sci Med. 2020;250:112852. doi: 10.1016/j.socscimed.2020.112852 32135459 PMC7429294

[12] Knaak S, Mantler E, Szeto A. Mental illness-related stigma in healthcare: Barriers to access and care and evidence-based solutions. Healthc Manage Forum. 2017;30(2):111–6. doi: 10.1177/0840470416679413 28929889 PMC5347358

[13] Gurung D, Subedi B, Kohrt BA, Wahid SS, Rai S, Thornicroft G, et al. Development of a mental health-related structural stigma measurement framework in the healthcare system setting: A modified Delphi study. PLoS One. 2025;20(1):e0316999. doi: 10.1371/journal.pone.0316999 39888878 PMC11785284

[14] Jordans MJD, Luitel NP, Kohrt BA, Rathod SD, Garman EC, De Silva M, et al. Community-, facility-, and individual-level outcomes of a district mental healthcare plan in a low-resource setting in Nepal: A population-based evaluation. PLoS Med. 2019;16(2):e1002748. doi: 10.1371/journal.pmed.1002748 30763321 PMC6375569

[15] Roberts T, Miguel Esponda G, Torre C, Pillai P, Cohen A, Burgess RA. Reconceptualising the treatment gap for common mental disorders: a fork in the road for global mental health? Br J Psychiatry. 2022;221(3):553–7. doi: 10.1192/bjp.2021.221 35137680

[16] Gronholm PC, Bakolis I, Cherian AV, Davies K, Evans-Lacko S, Girma E, et al. Toward a multi-level strategy to reduce stigma in global mental health: overview protocol of the Indigo Partnership to develop and test interventions in low- and middle-income countries. Int J Ment Health Syst. 2023;17(1):2. doi: 10.1186/s13033-022-00564-5 36732828 PMC9896727

[17] Clay J, Eaton J, Gronholm PC, Semrau M, Votruba N. Core components of mental health stigma reduction interventions in low- and middle-income countries: a systematic review. Epidemiol Psychiatr Sci. 2020;29:e164. doi: 10.1017/S2045796020000797 32883399 PMC7503169

[18] Rai S, Gurung D, Kohrt B. The PhotoVoice method for collaborating with people with lived experience of mental health conditions to strengthen mental health services. Glob Ment Health (Camb). 2023;10:e80. doi: 10.1017/gmh.2023.73 38161746 PMC10755382

[19] Semrau M, Gronholm PC, Eaton J, Maulik PK, Ayele B, Bakolis I, et al. Reducing stigma and improving access to care for people with mental health conditions in the community: protocol for a multi-site feasibility intervention study (Indigo-Local). Int J Ment Health Syst. 2024;18(1):35. doi: 10.1186/s13033-024-00649-3 39558379 PMC11575452

[20] Subba P, Luitel NP, Kohrt BA, Jordans MJD. Improving detection of mental health problems in community settings in Nepal: development and pilot testing of the community informant detection tool. Confl Health. 2017;11:28. doi: 10.1186/s13031-017-0132-y 29181088 PMC5694900

[21] Rai S, Gurung D, Kaiser BN, Sikkema KJ, Dhakal M, Bhardwaj A, et al. A service user co-facilitated intervention to reduce mental illness stigma among primary healthcare workers: Utilizing perspectives of family members and caregivers. Fam Syst Health. 2018;36(2):198–209. doi: 10.1037/fsh0000338 29902036 PMC6005191

[22] Gurung D, Kohrt BA, Wahid SS, Bhattarai K, Acharya B, Askri F, et al. Adapting and piloting a social contact-based intervention to reduce mental health stigma among primary care providers: Protocol for a multi-site feasibility study. SSM - Mental Health. 2023;4:100253. doi: 10.1016/j.ssmmh.2023.100253

[23] Kohrt BA, Turner EL, Gurung D, Wang X, Neupane M, Luitel NP, et al. Implementation strategy in collaboration with people with lived experience of mental illness to reduce stigma among primary care providers in Nepal (RESHAPE): protocol for a type 3 hybrid implementation effectiveness cluster randomized controlled trial. Implement Sci. 2022;17(1):39. doi: 10.1186/s13012-022-01202-x 35710491 PMC9205129

[24] Henderson C, Ouali U, Bakolis I, Berbeche N, Bhattarai K, Brohan E, et al. Training for mental health professionals in responding to experienced and anticipated mental health-related discrimination (READ-MH): protocol for an international multisite feasibility study. Pilot Feasibility Stud. 2022;8(1):257. doi: 10.1186/s40814-022-01208-8 36514144 PMC9745687

[25] Kohrt BA, Pedersen GA, Schafer A, Carswell K, Rupp F, Jordans MJD, et al. Competency-based training and supervision: development of the WHO-UNICEF Ensuring Quality in Psychosocial and Mental Health Care (EQUIP) initiative. Lancet Psychiatry. 2025;12(1):67–80. doi: 10.1016/S2215-0366(24)00183-4 39250925 PMC12056314

[26] Evans-Lacko S, Little K, Meltzer H, Rose D, Rhydderch D, Henderson C, et al. Development and psychometric properties of the Mental Health Knowledge Schedule. Can J Psychiatry. 2010;55(7):440–8. doi: 10.1177/070674371005500707 20704771

[27] Evans-Lacko S, Rose D, Little K, Flach C, Rhydderch D, Henderson C, et al. Development and psychometric properties of the reported and intended behaviour scale (RIBS): a stigma-related behaviour measure. Epidemiol Psychiatr Sci. 2011;20(3):263–71. doi: 10.1017/s2045796011000308 21922969

[28] Kiger ME, Varpio L. Thematic analysis of qualitative data: AMEE Guide No. 131. Med Teach. 2020;42:846–54. doi: 10.1080/0142159X.2020.1755030 32356468

[29] Saunders B, Sim J, Kingstone T, Baker S, Waterfield J, Bartlam B, et al. Saturation in qualitative research: exploring its conceptualization and operationalization. Qual Quant. 2018;52(4):1893–907. doi: 10.1007/s11135-017-0574-8 29937585 PMC5993836

[30] Rahimi S, Khatooni M. Saturation in qualitative research: An evolutionary concept analysis. Int J Nurs Stud Adv. 2024;6:100174. doi: 10.1016/j.ijnsa.2024.100174 38746797 PMC11080421

[31] Corrigan PW, Morris SB, Michaels PJ, Rafacz JD, Rüsch N. Challenging the public stigma of mental illness: a meta-analysis of outcome studies. Psychiatr Serv. 2012;63(10):963–73. doi: 10.1176/appi.ps.201100529 23032675

[32] Mendon GB, Gurung D, Loganathan S, Abayneh S, Zhang W, Kohrt BA, et al. Establishing partnerships with people with lived experience of mental illness for stigma reduction in low- and middle-income settings. Glob Ment Health (Camb). 2024;11:e70. doi: 10.1017/gmh.2024.69 39257677 PMC11383975

[33] Kohrt BA, Jordans MJD, Turner EL, Rai S, Gurung D, Dhakal M, et al. Collaboration With People With Lived Experience of Mental Illness to Reduce Stigma and Improve Primary Care Services: A Pilot Cluster Randomized Clinical Trial. JAMA Netw Open. 2021;4(11):e2131475. doi: 10.1001/jamanetworkopen.2021.31475 34730821 PMC8567115

[34] Sarikhani Y, Bastani P, Rafiee M, Kavosi Z, Ravangard R. Key barriers to the provision and utilization of mental health services in low-and middle-income countries: A scope study. Community Ment Health J. 2021;57:836–52. doi: 10.1007/s10597-020-00619-2 32285371

[35] Luitel NP, Jordans MJ, Adhikari A, Upadhaya N, Hanlon C, Lund C, et al. Mental health care in Nepal: current situation and challenges for development of a district mental health care plan. Confl Health. 2015;9:3. doi: 10.1186/s13031-014-0030-5 25694792 PMC4331482

[36] Rathod S, Pinninti N, Irfan M, Gorczynski P, Rathod P, Gega L. Mental Health Service Provision in Low- and Middle-Income Countries. 2017. 10.1177/1178632917694350 PMC5398308 28469456

[37] Stangl AL, Earnshaw VA, Logie CH, van Brakel W, C Simbayi L, Barré I, et al. The Health Stigma and Discrimination Framework: a global, crosscutting framework to inform research, intervention development, and policy on health-related stigmas. BMC Med. 2019;17(1):31. doi: 10.1186/s12916-019-1271-3 30764826 PMC6376797

[38] Gurung D, Subedi B, Acharya B, Neupane M, Kohrt BA, Thornicroft G, et al. Feasibility and Applicability of Implementing the Framework for Comprehensive Understanding of Structural Stigma in Mental Healthcare Systems: A Case Example of Nepal. Health Expect. 2025;28(1):e70170. doi: 10.1111/hex.70170 39910907 PMC11799572

[39] Zhamaliyeva L, Ablakimova N, Batyrova A, Veklenko G, Grjibovski AM, Kudaibergenova S, et al. Interventions to Reduce Mental Health Stigma Among Health Care Professionals in Primary Health Care: A Systematic Review and Meta-Analysis. Int J Environ Res Public Health. 2025;22(9):1441. doi: 10.3390/ijerph22091441 41007584 PMC12469428

[40] Zhang W, Henderson C, Magnusdottir E, Chen W, Ma N, Ma H, et al. Effect of a contact-based education intervention on reducing stigma among community health and care staff in Beijing, China: Pilot randomized controlled study. Asian J Psychiatr. 2022;73:103096. doi: 10.1016/j.ajp.2022.103096 35430494

