# Supplementary material for: Multi-level mental health-related stigma interventions in healthcare systems through the involvement of people with lived experiences: Mixed-methods pilot findings from international study of discrimination and stigma outcomes (INDIGO) study in Nepal
Source: PLOS Ment Health. 2026 Sep 8;3(9):e0000638. doi: 10.1371/journal.pmen.0000638 (PMC13552732; doi:10.1371/journal.pmen.0000638)
Supplement: Supplementary file 1 [file pmen.0000638.s001.docx]

**Additional file 1: The consolidated criteria for reporting qualitative research (COREQ)**

Tong A, Sainsbury P, Craig J. Consolidated criteria for reporting qualitative research (COREQ): a 32-item checklist for interviews and focus groups. International Journal for Quality in Health Care. 2007. Volume 19, Number 6: pp. 349 – 357

| **Domain 1: Research team and reflexivity** |  | | Reported on page no | |
| --- | --- | --- | --- | --- |
| **Personal Characteristics** | | | | |
| 1. Interviewer/facilitator Which author/s conducted the interview or focus group? | BS, KB and DG conducted the interviews | | 11 | |
| 2. Credentials  What were the researcher’s credentials? E.g. PhD, MD | KB was Bsc Biology graduate  BS was a BPH graduate  DG was a PhD fellow | | 11 | |
| 3. Occupation  What was their occupation at the time of the study? | KB and BS were research officers  DG was a countryside lead and project manager | | 11 | |
| 4. Gender  Was the researcher male or female? | Three females | | 11 | |
| 5. Experience and training  What experience or training did the researcher have? | DG had previous experiences of conducting, analyzing, and publishing qualitative research on mental health  BS and KB had taken training and workshop on the qualitative methods. | | 11 | |
| **Relationship with participants** | | | | |
| 6. Relationship established  Was a relationship established prior to study commencement? | No | | N/A | |
| 7. Participant knowledge of the interviewer  What did the participants know about the researcher? e.g. personal goals, reasons for doing the research | Participants were informed of the study's purpose and aims, and of the researchers' affiliations, prior to the interviews. | | N/A | |
| 8. Interviewer characteristics What characteristics were reported about the interviewer/facilitator? e.g. Bias, assumptions, reasons and interests in the research topic |  | | N/A | |
| **Domain 2: study design** | | | | |
| **Theoretical framework** | | | | |
| 9. Methodological orientation and Theory  What methodological orientation was stated to underpin the study? e.g. grounded theory, discourse analysis, ethnography, phenomenology, content analysis | Inductive and deductive coding with thematic analysis. | | 11-12 | |
| Participant selection | | | | |
| 10. Sampling  How were participants selected? e.g. | The participants were selected based on their availability during the interview time period. | | 8 | |
| 11. Method of approach  How were participants approached? e.g. face-to-face, telephone, mail, email | The participants were approached through telephone | | 8 | |
| 12. Sample size  How many participants were in the study? | 39 | | 11-12 | |
| 13. Non-participation  How many people refused to participate or dropped out? Reasons? | All of the respondents were invited for focus interviews. 4 were unable to participate due to their time constraints. | | 11 | |
| **Setting** | | | | |
| 14. Setting of data collection  Where was the data collected? e.g. home, clinic, workplace | | The data was collected at the feasible locations, to participants such as the local community hall, inside the hospital in a vacant room. | | 11 |
| 15. Presence of non-participants  Was anyone else present besides the participants and researchers? | | Only the interviewer and interviewee were presented inside the room | | 11 |
| 16. Description of sample  What are the important characteristics of the sample? e.g. demographic data, date | | The sample is described in table 1 | | 12-13 |
| **Data collection** | | | | |
| 17. Interview guide  Were questions, prompts, guides provided by the authors? Was it pilot tested? | | We used an interview guide based which was developed on the coding framework. | | 11 |
| 18. Repeat interviews  Were repeat interviews carried out? If yes, how many? | | No, interviews weren’t repeated | | N/A |
| 19. Audio/visual recording  Did the research use audio or visual recording to collect the data? | | The interviews were audio recorded. | | 11 |
| 20. Field notes  Were field notes made during and/or after the interview or focus group? | | The debriefing form were filled up to reflect upon the interviews by the interviewer. | | 11 |
| 21. Duration  What was the duration of the interviews or focus group? | | The duration was between 30-60 minutes | | 11 |
| 22. Data saturation  Was data saturation discussed? | | Yes, the data saturation was discussed for inductive coding to generate any new emerged themes. IT was agreed that now new themes were emerged by the end of the process. | | 11-12 |
| 23. Transcripts returned  Were transcripts returned to participants for comment and/or correction? | | No, the transcripts weren’t returned to the participants for comment or correction | | N/A |
| **Domain 3: analysis and findings** | | | | |
| **Data analysis** | | | | |
| 24. Number of data coders  How many data coders coded the data? | | BS, DG and KB coded the data; three coders | | 11-12 |
| 25. Description of the coding tree  Did authors provide a description of the coding tree? | | Yes. The coding process is described in data management section | | 11-12 |
| 26. Derivation of themes  Were themes identified in advance or derived from the data? | | Deductive and Inductive methods were used | | 11-12 |
| 27. Software What software, if applicable, was used to manage the data? | | Dedoose software | | 12 |
| 28. Participant checking  Did participants provide feedback on the findings? | | No, the participants didn’t provide feedback on the findings. | | N/A |
| **Reporting** | | | | |
| 29. Quotations presented Were participant quotations presented to illustrate the themes / findings? Was each  quotation identified? e.g. participant number | | Yes, quotes supporting the specific findings were attributed | | 13-20 |
| 30. Data and findings consistent  Was there consistency between the data presented and the findings? | | Yes | | 13-20 |
| 31. Clarity of major themes  Were major themes clearly presented in the findings? | | Yes | | 13-20 |
| 32. Clarity of minor themes  Is there a description of diverse cases or discussion of minor themes? | | No | | N/A |
